# Serum Methionine Metabolites Are Risk Factors for Metastatic Prostate Cancer Progression

**DOI:** 10.1371/journal.pone.0022486

**Published:** 2011-08-10

**Authors:** Sally Stabler, Tatsuki Koyama, Zhiguo Zhao, Magaly Martinez-Ferrer, Robert H. Allen, Zigmund Luka, Lioudmila V. Loukachevitch, Peter E. Clark, Conrad Wagner, Neil A. Bhowmick

**Affiliations:** 1 Department of Medicine, University of Colorado, Aurora, Colorado, United States of America; 2 Department of Biostatistics, Vanderbilt University, Nashville, Tennessee, United States of America; 3 Department of Surgery, University of Puerto Rico, San Juan, Puerto Rico; 4 Department of Biochemistry, Vanderbilt University, Nashville, Tennessee, United States of America; 5 Department Urologic Surgery, Vanderbilt University, Nashville, Tennessee, United States of America; 6 Department of Medicine, Cedars-Sinai Medical Center, Los Angeles, California, United States of America; University of Kentucky, United States of America

## Abstract

**Background:**

Clinical decision for primary treatment for prostate cancer is dictated by variables with insufficient specificity. Early detection of prostate cancer likely to develop rapid recurrence could support neo-adjuvant therapeutics and adjuvant options prior to frank biochemical recurrence. This study compared markers in serum and urine of patients with rapidly recurrent prostate cancer to recurrence-free patients after radical prostatectomy. Based on previous identification of urinary sarcosine as a metastatic marker, we tested whether methionine metabolites in urine and serum could serve as pre-surgical markers for aggressive disease.

**Methodology/Principal Findings:**

Urine and serum samples (n = 54 and 58, respectively), collected at the time of prostatectomy were divided into subjects who developed biochemical recurrence within 2 years and those who remained recurrence-free after 5 years. Multiple methionine metabolites were measured in urine and serum by GC-MS. The role of serum metabolites and clinical variables (biopsy Gleason grade, clinical stage, serum prostate specific antigen [PSA]) on biochemical recurrence prediction were evaluated. Urinary sarcosine and cysteine levels were significantly higher (p = 0.03 and p = 0.007 respectively) in the recurrent group. However, in serum, concentrations of homocysteine (p = 0.003), cystathionine (p = 0.007) and cysteine (p<0.001) were more abundant in the recurrent population. The inclusion of serum cysteine to a model with PSA and biopsy Gleason grade improved prediction over the clinical variables alone (p<0.001).

**Conclusions:**

Higher serum homocysteine, cystathionine, and cysteine concentrations independently predicted risk of early biochemical recurrence and aggressiveness of disease in a nested case control study. The methionine metabolites further supplemented known clinical variables to provide superior sensitivity and specificity in multivariable prediction models for rapid biochemical recurrence following prostatectomy.

## Introduction

Prostate cancer remains the most common non-cutaneous solid malignancy in the United States, and the second leading cause of cancer specific death in men. Nevertheless, it has become increasingly clear that not all men who are diagnosed with prostate cancer require intervention [1]. Yet, many men that receive surgical or radiation-based primary treatment develop recurrent disease. Prior to surgical intervention, serum PSA, biopsy Gleason grade, and clinical stage help determine if patients are likely to recurred versus those that may remain localized and possibly remain clinically inconsequential. Various approaches in improving the role of PSA in early prostate cancer detection have been tested, but their benefit to overall survival is yet to be proven [2], [3]. Ultimately, there is a subgroup of men without conventional negative factors harboring high risk, aggressive disease and are even at elevated risk of early recurrence after attempted definitive local therapy [4], [5], [6]. The ongoing challenge facing clinicians is how to identify this cohort of men at high risk, from the larger cohort of men who are likely harboring more indolent disease [7]. New markers of aggressive disease are therefore needed for an informed clinical decision.

A previous study identified sarcosine (N-methylglycine) as a product of methionine catabolism that is elevated in the urine of patients with metastatic prostate disease [8]. Sarcosine levels were higher in tissues from localized prostate cancer than in normal tissue, and even higher in metastatic prostate tissue. Urinary sarcosine was thus suggested as a possible marker for metastatic prostate cancer. The enzyme, Glycine N-methyltransferase (GNMT) is the primary source of sarcosine in liver, where it accounts for about 1% of the soluble protein [9]. Individuals with defective sarcosine dehydrogenase have sarcosinemia, but show no distinctive phenotype [10]. However, a reported causative role for sarcosine in prostate cancer metastasis [8], suggests therapeutic targeting of its metabolic pathway to be useful.

In this study we evaluated the serum and urine of radical prostatectomy patients for metabolites to differentiate those who developed early biochemical recurrence (rise in serum PSA≥0.2 ng/ml) within two years of surgery and those who remained recurrence-free after more than five years. We found that the urine of patients in the rapidly recurrent group had significantly higher concentrations of sarcosine and cysteine than those in the recurrence-free group. In addition, significantly greater concentrations of serum cystathionine, homocysteine and cysteine were found in the rapidly recurrence group compared to the recurrence-free group. These products of elevated methionine catabolism in patients with rapidly recurrent prostate cancer represent pre-surgical indicators that augmented serum PSA for the prediction of clinically significant prostate cancer.

## Methods

### Ethics Statement

This nested case-control study was conducted in accordance with the Institutional Review Board of Vanderbilt University. Written consent was given by the patients for their information to be stored in the hospital database. The board specifically approved the research use of the di-identified information and “on the shelf” specimens to be used for research under a waiver of consent.

### Patient selection

The digital medical records of 400 subjects were retrospectively examined using the Vanderbilt University Department of Urologic Surgery registry of radical prostatectomies performed between 2003 and 2007. Several patients were excluded for reasons of compromised renal, heart, or liver function as was determined by electronic records of elevated urinary creatinine, hypertension, cardiac infarction history, and blood markers for hepatic function. Additionally, availability of follow-up data and records of pre-surgical hormone-ablation therapy were reasons for exclusion. Rapidly recurrent patients were identified as those who developed biochemical recurrence following prostatectomy within 2 years (American Joint Committee on Cancer defined as having PSA≥0.2 ng/ml, confirmed at least once two weeks later). The recurrence-free population was defined as having maintained a serum PSA<0.01 ng/ml for five or more years following surgery. Ultimately, for this nested case control study we focused on 54 subjects for analysis of urine and 58 subjects for analysis of serum who developed rapid biochemical recurrence and an age-matched recurrence-free control group who were free of recurrence. The mean age for the subjects was 60 years. All subjects were annotated based on age, pre-surgical serum PSA, biopsy Gleason score, clinical stage, and detection of biochemical recurrence.

### Urine and Serum Quantitative Metabolic Analysis

Serum and urine obtained at the time of radical prostatectomy were rapidly processed and stored at −80°C. We evaluated serum and urine for the metabolites, sarcosine, dimethylglycine, methionine, homocysteine, cystathionine, cysteine, methylmalonic acid and methylcitrate by gas-liquid chromatography/mass spectrometry [11], [12], [13]. Folate was measured microbiologically as described by Horne [14]. Urinary metabolites were expressed as nmol/mg creatinine to correct for differences in urine volume. Creatinine in urine was measured by the Jaffe method [15].

### Statistical Analysis

Patients' baseline demographic and clinical variables were assessed using Wilcoxon rank sum tests for continuous variables and Fisher exact tests for categorical (including binary) variables. All marker values, as well as PSA levels, were logarithmically transformed to achieve normality. Correlations among the markers were assessed using Spearman's rank correlation. Logistic regression models were used to analyze incidence of recurrence. The base model includes serum PSA, biopsy Gleason score, and clinical stage, clinical variables that are available prior to surgery. The post-surgical variables (e.g., lymph nodes, surgical margins, pathologic Gleason scores) were not considered. For multiplicity control, p≤0.007 (p-value less than 5%/7 = 0.7%) was considered statistically significant. To avoid further overfitting of the data, no variable selection was performed in the subsequent analyses based on logistic regression models. We used a likelihood ratio test to compare the simpler model (without the metabolites) and the full model (with the individual metabolites). Receiver operating characteristics (ROC) curves were generated for each logistic regression model, where the area under ROC curve (AUC) was determined. Integrated discrimination improvement (IDI) and Net reclassification index (NRI) [16] were used to compare the models' ability to distinguish recurrence and non-recurrence. The logrank tests were used to assess the difference in recurrence-free survival between the two groups illustrated by Kaplan-Meier plots. For the selected markers, Cox proportional hazard regression models were fit, and likelihood ratio tests were used to assess markers' association with time to recurrence outcome. The proportional hazard assumption was assessed using the method of Grambsch and Therneau [17]. All data analyses were performed using R 2.10.1 (R Development Core Team, Vienna, Austria); a significance level of 0.05 was used for statistical inference unless otherwise noted.

## Results

### Methionine metabolites support prediction of biochemical recurrence

Urine metabolites were initially measured in fifty-four patients who developed biochemical recurrence (N = 25) and those that remained recurrence-free (N = 29). These patients were matched for age and pre-surgical serum PSA. **Table 1** enumerates the clinical characteristics of the two patient groups by serum PSA, clinical stage, and biopsy Gleason grade. Majority of patients had a clinical stage of T1. Creatinine-normalized urinary dimethylglycine and homocysteine were not significantly different between the two groups. However, we found urinary sarcosine to be significantly elevated at the time of surgery in patients who developed biochemical recurrence, as originally reported for patients with frank prostate metastatic lesions [8]. We further found that urinary cysteine was significantly elevated in biochemically-recurrent patients compared to those who remained recurrence-free five years following prostatectomy. Urine analysis in a pre-surgical patient population suggested products of methionine catabolism might correlate with prostate cancer progression status.

**Table 1:** The values for methionine metabolites measured in the urine of the recurrent-free and the recurrent groups are compared.

	Recurrent-free (29)	Recurrent (25)	P value
**Age**	59 (53, 64)	62 (58, 67)	0.10
**Pre-surgery PSA**	5.2 (4.3, 6.5)	6.0 (5.0, 8.2)	0.08
**Clinical stage** (N = 16/18)			0.09
**T1**	15 (94%)	12 (67%)	
**T2**	1 (6%)	6 (33%)	
**T3**	0	0	
**Biopsy Gleason** (N = 16/18)			0.050
**4**	1 (6%)	0	
**5**	2 (12%)	0	
**6**	9 (56%)	4 (22%)	
**7**	3 (19%)	8 (44%)	
**8**	1 (6%)	3 (17%)	
**9**	0	2 (11%)	
**10**	0	1 (6%)	
**Urine cysteine** (N = 29/24)	190 (168, 212)	221 (189, 252)	0.007
**Urine homocysteine**	2.7 (2.2, 3. 2)	2.8 (2.4, 4.0)	0.40
**Urine dimethylglycine**	27.3 (22.1, 38.5)	25.4 (17.6, 33.7)	0.34
**Urine sarcosine**	3.7 (3.1, 5.7)	5.4 (4.1, 6.7)	0.03

Values for sarcosine, homocysteine, dimethylglycine and cysteine are expressed as µmoles/mg creatinine. Wilcoxon rank sum tests for continuous variables and Fisher exact tests for categorical (including binary) variables are indicated. Normal values for metabolites (nmole/mg creatinine) are: cysteine, 140–579; homocysteine, 0.974–7.17; dimethylglycine, 10.1–108.2 and sarcosine, 2.65–8.67. Median values with quartiles were used to summarize the distributions of the continuous variables.

We then performed a nested case control study with pre-surgical serum. Fifty-eight age-matched prostatectomy patients were stratified by pre-surgical PSA, clinical stage, and biopsy Gleason grade as well as pathologic variables (**Table 2**). As expected, clinical variables were significantly different in the two populations, as were the post-surgical pathologic factors. Interestingly, the serum homocysteine, cystathionine, and cysteine were significantly higher in the biochemically-recurrent patients (p value<0.001). However, clinical stage and serum levels of sarcosine, dimethylglycine, folate, methylcitrate, and methylmalonic acid were not significantly different between the two populations. Normal methylcitrate levels in both populations supported renal sufficiency. Serum methylmalonic acid levels, an indicator of vitamin B-12 status [18], were not different between the two groups. Serum and urine cysteine correlation did not reach statistical significance (p = 0.06, **Table 3**). However, serum homocysteine was strongly correlated with cysteine (Spearman's rank correlation = 0.65, p<0.01). Therefore, the higher serum homocysteine was not a function of differences in renal function, vitamin B-12 or folate status.

**Table 2:** The values for methionine metabolites measured in the sera of the recurrent-free and the recurrent groups are compared.

	Recurrent-free (30)	Recurrent>(28)	P value
**Age**	59 (54, 64)	61 (59, 64)	0.07
**Pre-surgery PSA**	5.4 (4.0, 8.1)	6.8 (5.2, 8.9)	0.02
**Clinical stage**			0.30
**T1**	24 (80%)	18 (64%)	
**T2**	6 (20%)	9 (32%)	
**T3**	0	1 (4%)	
**Biopsy Gleason**			0.006
**4**	1 (3%)	0	
**5**	2 (7%)	0	
**6**	18 (60%)	6 (20%)	
**7**	6 (20%)	13 (46%)	
**8**	2 (7%)	4 (15%)	
**9**	1 (3%)	4 (15%)	
**10**	0	1 (4%)	
**Serum cysteine**	346 (321, 377)	419 (367, 452)	<0.001
**Serum homocysteine**	9.0 (8.0, 10.2)	11.7 (9.4, 13.4)	0.003
**Serum dimethylglycine** (n = 27/23)	4.6 (3.8, 4.7)	4.9 (4.2, 5.4)	0.21
**Serum sarcosine** (n = 27/23)	1.3 (1.1, 1.4)	1.3 (1.1, 1.7)	0.67
**Serum methionine** (n = 27/27)	24.8 (21.7, 30.6)	27.6 (23.9, 33.7)	0.08
**Serum folate** (n = 27/28)	44.8 (25.2, 52.8)	42.3 (31.3, 51.5)	0.72
**Serum methylcitrate**	126 (102, 144)	135 (117, 167)	0.13
**Serum methylmalonate**	167 (145, 220)	164 (146, 211)	0.91
**Serum cystathionine** (n = 29/26)	149 (130, 176)	186 (148, 239)	0.007
**Lymph node involvement**	0 (0%)	6 (21%)	0.01
**SV involvement**	0 (0%)	8 (29%)	0.002
**Positive surgical margin**	1 (3%)	8 (29%)	0.01
**Stage III+**	3 (10%)	21 (75%)	<0.001
**Pathologic Gleason**			0.002
**5**	2 (7%)	0 (0%)	
**6**	15 (50%)	4 (14%)	
**7**	10 (33%)	14 (50%)	
**8**	3 (10%)	4 (14%)	
**9**	0 (0%)	6 (21%)	

Wilcoxon rank sum tests for continuous variables and Fisher exact tests for categorical (including binary) variables are indicated. Normal values for metabolites are: cysteine, 203–369 µM homocysteine, 5.4–13.9 µM; dimethylglycine, 1.4–5.3 µM; sarcosine, 0.6–2.7 µM; methionine, 11.3–42.7 µM; folate, >3.0 ng/ml; methylcitrate, 60–228 nM; methylmalonate, 73–271 nM; cystathionine, 44–342 nM. Median values with quartiles were used to summarize the distributions of the continuous variables.

**Table 3:** Correlations between serum and urine markers.

	Correlation coefficient	P value	n
**Sarcosine**	0.19	0.34	28
**Dimethylglycine**	0.12	0.53	28
**Cysteine**	0.33	0.06	33
**Homocysteine**	0.13	0.48	34

All correlations are rank based “Spearman's rho”.

**Figure 1:**
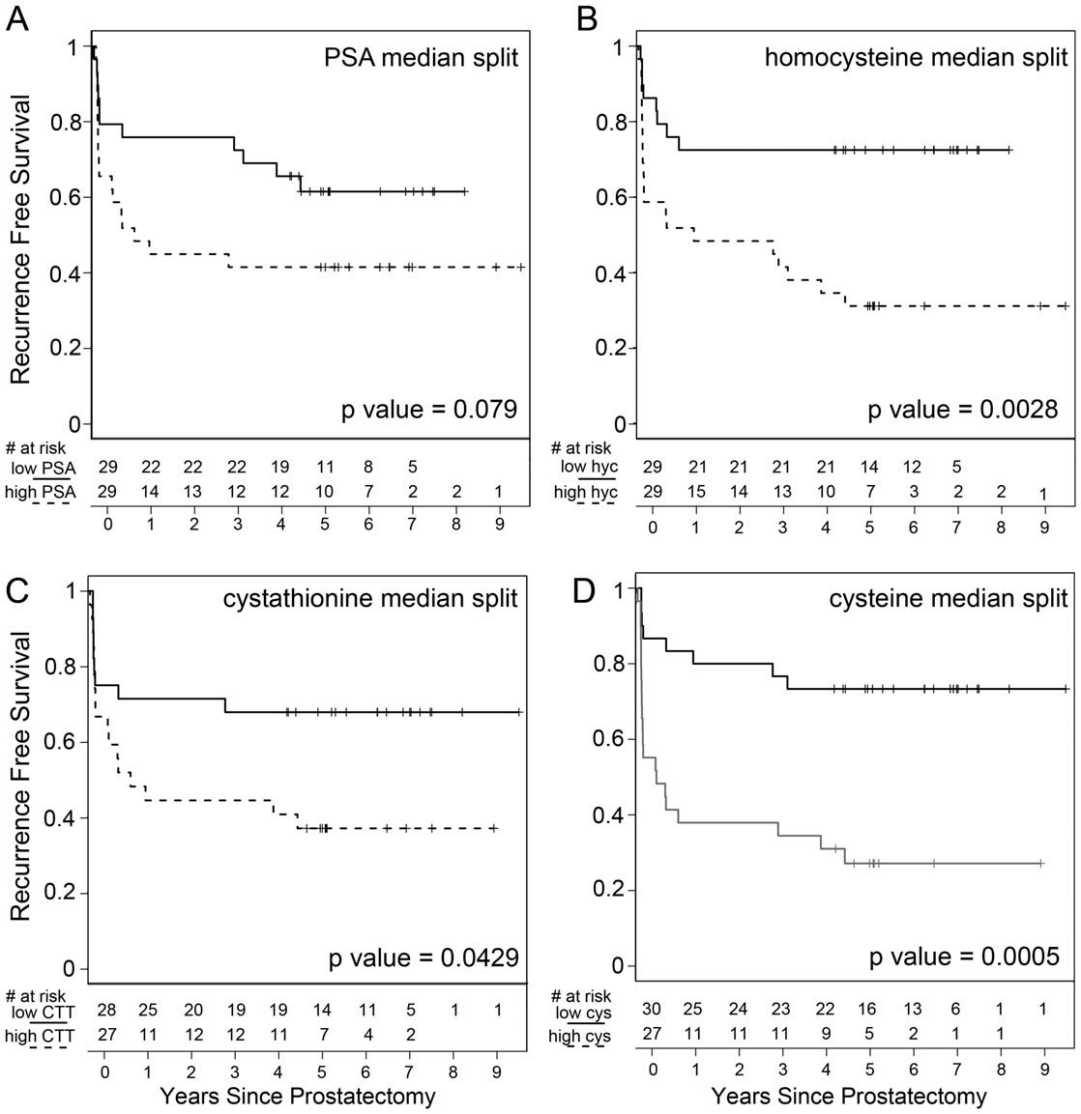
Kaplan-Meier plots indicate univariate predictive values of the recurrence-free survival based on pre-surgical serum. The patients were separated into two groups, divided at median tissue level for (A) PSA, (B) homocysteine, (C) cystathionine, and (D) cysteine as significantly associated with time to recurrence (Table 5). Those subjects above the median expression level were termed upper half, whereas those below the median were termed lower half. The recurrence-free survival probabilities were estimated by the Kaplan-Meier method and the differences were tested using the log-rank test. Each of the dichotomous serum markers supported statistically significant differences in biochemical recurrence-free survival.

The relevance of these newly identified markers to patient recurrence status were illustrated in Kaplan-Meier plots for homocysteine, cystathionine, and cysteine as compared to pre-operative serum PSA levels, and time-to-recurrence (**Figure 1**). Each of the markers could separate rapidly recurrent from the recurrence-free progression. However, serum cysteine detection had the greatest discriminatory power in the two populations prior to prostatectomy.

**Figure 2:**
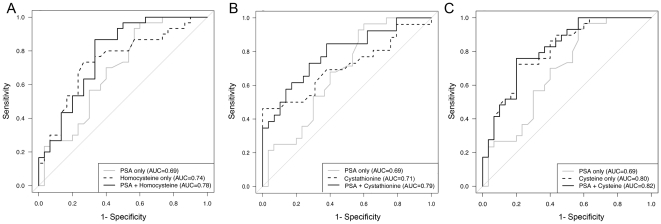
Receiver Operator Curve (ROC) for a statistical model that can be used to predict recurrence of prostate cancer based on serum derived variables. Serum PSA is compared to the added value of serum (A) homocysteine, (B) cystathionine, and (C) cysteine. In the ROC curve the probability with greater Area Under the Curve (AUC) support increased specificity and sensitivity over random guess, represented by the dotted diagonal line.

The clinical value of these methionine metabolites as biomarkers would be to significantly increase the ability to predict aggressive prostate cancer features and early biochemical recurrence over and above existent clinical variables including serum PSA, biopsy Gleason score, and clinical stage. We developed a multiple logistic regression model for the prediction of biochemical recurrence based on serum methionine metabolites and the pre-surgical predictor variables, serum PSA and biopsy Gleason grade. Since majority of patients in both cohorts had clinical stage T1c disease, this variable had little discriminative power and was dropped from the model. Serum cysteine, cystathionine, and homocysteine were the top three predictors for recurrence in 70% of the patients, so further analysis of methionine metabolites focused on these three metabolites. Correlations between cysteine and homocysteine were the highest among all pair-wise correlations (R^2^ = 0.65, p<0.01), and cysteine was also highly correlated with cystathionine (R^2^ = 0.39, p<0.01, **Table 4**). Addition of serum homocysteine provided the greatest improvement of the logistic regression models compared to the base model with PSA and biopsy Gleason (p = 0.0007), followed by cysteine (p = 0.0017), and cystathionine (p = 0.0037). Correlation between cystathionine and homocysteine was moderate (R^2^ = 0.22, p = 0.10). Based on multiple logistic regression models (**Table 5**), odds of recurrence increased 5.79 fold (95% CI: 1.65 to 20.29, p = 0.006) when cysteine levels increased from 343 (lower quartile, henceforth Q1) to 436 (upper quartile, henceforth Q3). This logistic regression model did not find pre-surgical serum PSA levels to be significantly associated with recurrence status. In a separate model, cystathionine levels were significantly associated with recurrence status. Odds of recurrence were 2.44 (95% CI: 1.07 to 5.56, p = 0.03) times higher when cystathionine levels were increased from 139 (Q1) to 200 (Q3). Serum PSA levels were marginally associated with recurrence in this model; the odds ratio was 2.94 (95% CI: 1.02 to 8.48, p = 0.046) when PSA levels were increased from 4.7 (Q1) to 8.5 (Q3). Homocysteine levels were also found to be associated with recurrence status. In all of these models biopsy Gleason grade was significantly associated with recurrence. To evaluate the additional utility of these three markers, the models including cysteine, cystathionine, or homocysteine in addition to serum PSA levels and biopsy Gleason grade were compared to a model utilizing PSA plus biopsy Gleason only. Clinical stage values did not contribute to the improvement of the models. Area under the ROC curves were similar (AUC = 0.86) for the cysteine, cystathionine, and homocysteine when combined with the clinical variables and significantly superior to the clinical variables alone (AUC = 0.81). The Integrated Discrimination Improvement (IDI) and Net Reclassification Improvement (NRI) supported the statistical significance of the improvement (**Table 6**). The benefit of these metabolites as combined with the standard PSA test is evident when PSA sensitivity and specificity were compared to a combined prediction of biochemical recurrence by the ROC in **Figure 2** following prostatectomy, using only serum PSA. The AUC with only serum markers were similar to the more comprehensive ones including biopsy results. There was a significant association between these markers and recurrence status, however the markers did not necessarily indicate usefulness in predicting recurrence-free survival.

**Table 4:** Correlations among serum markers.

	Dimethylglycine	Sarcosine	Cysteine	Cystathionine
**Homocysteine**	0.28, 0.05 n = 50	0.28, 0.04 n = 50	0.65,<0.01 n = 57	0.22, 0.10 n = 55
**Dimethylglycine**		0.35, 0.01 n = 50	0.40, <0.01 n = 50	0.16, 0.26 n = 48
**Sarcosine**			0.35, <0.01 n = 50	0.08, 0.60 n = 48
**Cysteine**				0.39, <0.01 n = 54

All correlations are rank based “Spearman's rho”, presented as correlation, p-value, and n.

**Table 5:** Logistic regression models.

SERUM HOMOCYSTEINE MODEL				
Variable	Comparison Q3∶Q1	Odds	95% Confidence Int.	P value
Pre-surgery PSA	**8.5 ∶ 4.7**	**2.39**	**(0.90, 6.33)**	**0.080**
Biopsy GS	**7 ∶ 6**	**4.29**	**(1.59, 11.56)**	**0.004**
Serum homocysteine	**12.5 ∶ 8.6**	**4.74**	**(1.61, 13.90)**	**0.005**

SERUM CYSTATHIONINE MODEL				
Variable	Comparison Q3∶Q1	Odds	95% Confidence Int.	P value
Pre-surgery PSA	**8.5 ∶ 4.7**	**2.94**	**(1.02, 8.48)**	**0.046**
Biopsy GS	**7 ∶ 6**	**2.80**	**(1.24, 6.28)**	**0.013**
Serum cystathionine	**200 ∶ 139**	**2.44**	**(1.07, 5.56)**	**0.033**

SERUM CYSTEINE MODEL				
Variable	Comparison Q3∶Q1	Odds	95% Confidence Int.	P value
Pre-surgery PSA	**8.5 ∶ 4.7**	**1.82**	**(0.66, 4.96)**	**0.245**
Biopsy GS	**7 ∶ 6**	**2.51**	**(1.19, 5.31)**	**0.015**
Serum cysteine	**436 ∶ 343**	**5.79**	**(1.65, 20.29)**	**0.006**

**Table 6:** The Integrated Discrimination Improvement (IDI) and Net Reclassification Improvement (NRI) were summarized below, supporting the statistical significance of the improvement.

	IDI	95% CI	P-value	NRI	95% CI	P-value
**Homocysteine**	0.14	0.05–0.24	0.003	1.03	0.52–1.55	<0.001
**Cystathionine**	0.12	0.004–0.20	0.003	0.81	0.28–1.34	0.003
**Cysteine**	0.14	0.04–0.23	0.005	0.64	0.13–1.16	0.015

To define the efficacy of the markers in predicting recurrence-free survival, Cox proportional hazard regression models were fit showing that cysteine, cystathionine, and homocysteine were each independent predictors of recurrence-free survival when adjusting for pre-operative serum PSA and biopsy Gleason score (**Table 7**). Specifically, serum cysteine, cystathionine, and homocysteine values increased (p<0.001, p = 0.014, p<0.001, respectively) with increased risk of recurrence on multivariable analysis with adjustment for both serum PSA and biopsy Gleason score.

**Table 7:** Cox regression models.

SERUM HOMOCYSTEINE MODEL				
Variable	Comparison Q3∶Q1	Hazard	95% Confidence Int.	P value
Pre-surgery PSA	**8.5 ∶ 4.7**	**2.34**	**(1.27, 4.32)**	**0.007**
Biopsy GS	**7 ∶ 6**	**2.01**	**(1.44, 2.79)**	**<0.001**
Serum homocysteine	**12.5 ∶ 8.6**	**2.43**	**(1.48, 4.01)**	**<0.001**

SERUM CYSTATHIONINE MODEL				
Variable	Comparison Q3∶Q1	Hazard	95% Confidence Int.	P value
Pre-surgery PSA	**8.5 ∶ 4.7**	**2.47**	**(1.30, 4.70)**	**0.006**
Biopsy GS	**7 ∶ 6**	**1.64**	**(1.21, 2.22)**	**0.001**
Serum cystathionine	**200 ∶ 139**	**1.69**	**(1.11, 2.57)**	**0.014**

SERUM CYSTEINE MODEL				
Variable	Comparison Q3∶Q1	Hazard	95% Confidence Int.	P value
Pre-surgery PSA	**8.5 ∶ 4.7**	**2.00**	**(1.03, 3.86)**	**0.039**
Biopsy GS	**7 ∶ 6**	**1.71**	**(1.24, 2.37)**	**0.001**
Serum cysteine	**436∶343**	**2.59**	**(1.51, 4.43)**	**<0.001**

## Discussion

Current risk stratification of patients prior to surgery involves variables including serum PSA, clinical stage, and biopsy grade. Independent serum markers in conjunction with PSA could help distinguish patients with aggressive prostate cancer. In the current era of PSA testing, clinical staging has reduced relevance when tumor volumes are relatively small. In our study, the highest biopsy Gleason score in ≥8-core biopsies provided a significant independent predictor comparable to serum cysteine and homocysteine. However, routine ultrasound directed first biopsies are reported to miss nearly a quarter of the prostate cancers [19] and often underestimate tumor grade [20], [21]. The combination of serum PSA with cystathionine, cysteine, and homocysteine as markers could improve decision-making for primary treatment and earlier subsequent adjuvant therapy.

**Figure 3:**
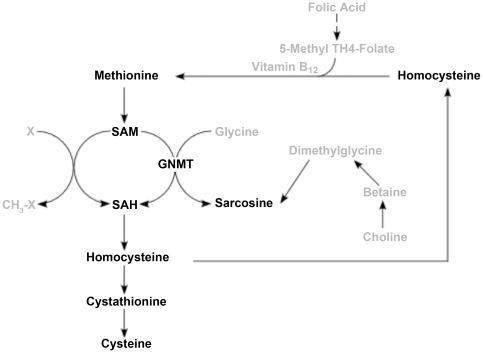
Methionine metabolism. Methionine is first converted to SAM, the donor of methyl groups in all but one methyltransferase reaction. SAM may transfer the methyl group to a variety of compounds, X, by a group of specific enzymes to yield the methylated compounds, CH_3_-X (eg. methylated lipids, DNA, or proteins). Alternatively, SAM may transfer the methyl group to glycine to form sarcosine via the enzyme glycine N-methyltransferase (GNMT. After transfer of the methyl group SAM is converted to S-adenosylhomocysteine (SAH), which is broken down further to homocysteine, cystathionine and cysteine. Sarcosine may also be formed by breakdown of choline to betaine, which, after loss of a methyl group, is converted to dimethylglycine. A dehydrogenase converts dimethylglycine to sarcosine.

Pathways of methionine metabolism involve two mechanisms for sarcosine formation (**Figure 3**). Cystathionine and cysteine are products of homocysteine catabolism important in production of glutathione. Elevation of urinary sarcosine in the absence of serum sarcosine differences was surprising, and likely the result of differential renal sarcosine excretion. Changes in sarcosine but not dimethylglycine suggest that increased activity of GNMT might have been present in the recurrent group. It is possible that for unknown reasons the recurrent group had increased S-adenosylmethionine (SAM) which activated the transulfuration pathway [22] thus, increasing cystathionine, cysteine, and formation of sarcosine. It should be noted that Sreekumar et al. [8] did not report sarcosine in patient serum or plasma associated with metastatic prostate cancer. Our data in pre-surgical subjects supports the previous report of urinary sarcosine elevation in confirmed metastatic patients. The data could mean that our patient population had previously undetected metastasis or that the elevated methionine metabolism is a precursor for metastasis. The direct role of sarcosine on metastatic progression is controversial. In contrast to the report of sarcosine directly supporting metastasis [8], a recent report suggests no association between urinary sarcosine levels and either tumor stage or Gleason score [23]. It is difficult to compare our findings with others reports since the initial study by Sreekumar et al [8] differ in the methodology of sarcosine measurement [24], sample source [25], [26], and importantly criteria defining recurrence [23]–[27]. Our assay utilizes a stable isotope internal standard in each sample, retrieved urine and serum prior to prostate resection, and recurrence was only based on serum PSA detection. Another study compared benign controls against patients with active prostate cancer and found that urine sarcosine was only a modest predictor of disease, but when added to other new markers such as prostate cancer antigen 3 and percent-free PSA improved diagnostic power [27]. There is abundant evidence that folate and B12 deficiency and kidney disease can contribute to hyperhomocysteinemia. However, in the present investigation there was no difference in folate or methylmalonic acid levels between recurrent and non-recurrent groups. The patients in this study were accordingly recruited to minimize complicating co-morbidities. The differences we found in homocysteine, cystathionine and cysteine in serum suggest that there may be systemic metabolic differences in those patients who go on to have a biochemical relapse.

The majority of the sarcosine produced in the body is made in the liver as a downstream product of SAM and homocysteine. Studies using homozygous mice with GNMT knocked out have plasma SAM levels 50% greater than that of wild type. The SAM levels in the livers of the *Gnmt* null animals were 33 fold higher than in the livers of wild type animals and all of the *Gnmt* null animals developed hepatocellular carcinoma after 8 months [28]. Interestingly, higher cysteine values are associated with obesity [29]–[32]. The limited body composition data for our subject groups, however suggested little correlation of body mass index and recurrence rate. The data reported here, support increased flux through GNMT resulting in the increased formation of homocysteine and sarcosine through increased utilization of SAM. Interestingly, GNMT, is reported to be down-regulated in neoplastic tissues in general [33] including human prostate cancer [34]. Thus, the changes seen in the current investigation may not be a result of neoplastic changes in prostate tissue. These results suggest the hypothesis that there may be differences in the methylation capacity of different individuals or tumor hosts as a result of different levels of SAM. Unfortunately, SAM values could not be measured in the current study, because of the instability of SAM in stored serum samples. Further, it is possible that individuals with a greater methylating capacity are more likely to develop cancer leading to metastatic progression.

To our knowledge, no previous study has correlated an entire arm of a metabolic pathway in the aggressiveness of cancer. In our study the comparison was made between patients with proven cancer, not between subjects with proven cancer and benign prostatic disease. These results were obtained with only a relatively small group of patients but the results are significant and suggest that further studies are needed. The underlying biology supports the robustness of these markers. Higher serum homocysteine, cystathionine, and cysteine improved the utility of currently used clinical variables in predicting early recurrence and suggest a greater flux of methyl groups through the enzyme GNMT.
